# An Explainable Machine Learning Approach Based on Statistical Indexes and SVM for Stress Detection in Automobile Drivers Using Electromyographic Signals

**DOI:** 10.3390/s21093155

**Published:** 2021-05-01

**Authors:** Olivia Vargas-Lopez, Carlos A. Perez-Ramirez, Martin Valtierra-Rodriguez, Jesus J. Yanez-Borjas, Juan P. Amezquita-Sanchez

**Affiliations:** 1 ENAP-Research Group, CA-Sistemas Dinámicos y Control, Facultad de Ingeniería, Campus San Juan del Río, Universidad Autónoma de Querétaro (UAQ), Río Moctezuma 249, San Juan del Rio 76807, Mexico; ovargas08@alumnos.uaq.mx (O.V.-L.); martin.valtierra@enap-rg.org (M.V.-R.); 2 ENAP-Research Group, Facultad de Ingeniería, Campus Aeropuerto, Universidad Autónoma de Querétaro (UAQ), Carretera a Chichimequillas S/N, Ejido Bolaños, Queretaro 76140, Mexico; carlos.perez@uaq.mx; 3 CA Procesamiento Digital de Señales, División de Ingenierías Campus Irapuato-Salamanca (DICIS), Universidad de Guanajuato, Salamanca 36885, Mexico; jjyanezb.ugto@gmail.com

**Keywords:** stress detection, EMG signals, statistical time features, support vector machine

## Abstract

The economic and personal consequences that a car accident generates for society have been increasing in recent years. One of the causes that can generate a car accident is the stress level the driver has; consequently, the detection of stress events is a highly desirable task. In this article, the efficacy that statistical time features (STFs), such as root mean square, mean, variance, and standard deviation, among others, can reach in detecting stress events using electromyographical signals in drivers is investigated, since they can measure subtle changes that a signal can have. The obtained results show that the variance and standard deviation coupled with a support vector machine classifier with a cubic kernel are effective for detecting stress events where an AUC of 0.97 is reached. In this sense, since SVM has different kernels that can be trained, they are used to find out which one has the best efficacy using the STFs as feature inputs and a training strategy; thus, information about model explain ability can be determined. The explainability of the machine learning algorithm allows generating a deeper comprehension about the model efficacy and what model should be selected depending on the features used to its development.

## 1. Introduction

Stress is defined as the reaction of the sympathetic nervous system to any type of threat, which generates a sudden release of hormones such as adrenaline and cortisol into the body [1]. These hormones induce to the body to a state of emergency or alert, which can cause an increase in the heart rate, muscle tension, increased blood pressure, accelerated breathing, and an increased acuity of the senses [2], among other reactions. Hence, this condition can negatively affect the daily life as well as the wellness of a person that experiences frequent stress events [3]. In particular, automobile drivers can be affected by this condition because of negative mood, lane departure, running red lights, traffic noise, congestion, heavy traffic, lack of sleep, driving phobia, impatience, curved narrow roads, and fatigue, among other causes [4,5,6,7,8], which can limit their concentration and ability to make reasonable decisions during any event. In consequence, stress, along with the abovementioned factors, could be an additional factor in certain car accidents that can inflict serious injuries upon those who are involved, even causing deaths [9,10]. In this regard, the World Health Organization reports that each year, about 1.35 million deaths are caused by road traffic crashes, where people from 5 to 29 years old are mainly involved [11]. Hence, it is of imperative importance to develop strategies or methodologies with the capability of detecting stress in drivers in a timely manner, allowing them to take preventive actions in order to avoid car accidents and injuries, which can negatively affect the driver’s life quality as well as that of the people involved in the accident.

**Figure 1:**
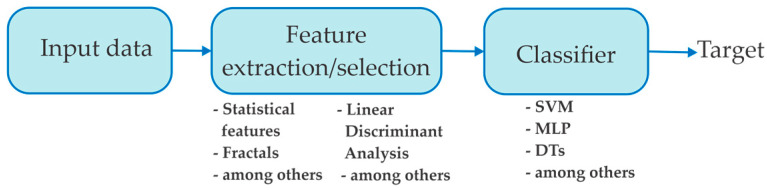
Machine learning framework for designing data-driven classifier schemes.

In recent years, machine learning algorithms have been used for detecting stress in drivers. This method has two main steps: (1) feature extraction and (2) classification or pattern recognition, as shown in Figure 1 [12,13,14,15,16,17,18,19]. For feature extraction, the measures of the physiological signals are extracted through different methods in order to find a particular pattern that can be associated with presence of stress events in drivers, so the classification algorithm of the extracted features are used for designing and training various algorithms that can automatically recognize stress in drivers [13]. In this sense, several researchers worldwide have presented different methods or methodologies for detecting stress in automobile drivers, which are based mainly on physiological signals such as electrocardiogram (ECG), galvanic skin response (GSR), electromyogram (EMG), or breathing rate, among others [14,15,16,17,18,19,20], using machine learning-based classifiers. For example, Munla et al. [14] developed a methodology based on wavelet transform; statistical machine learning (i.e., maximum, minimum, mean, among others) features of the heart rate variability (HRV) were obtained from ECG signals with a support vector machine (SVM) as classifier to detect whether the driver is stressed or not. The proposal used the information of 16 participants provided by the database *Stress Recognition in Automobile Drivers* (SRAD) [10], which includes recordings of ECG, GSR, and EMG signals. The authors reported that their proposal can determine when the driver suffers from stress with an accuracy of 83.33%. In this study, a preprocessing step is applied with the wavelet transform, which increases the computational load to the methodology. It should be noted that the following papers reviewed in this section use the same database. Rizwan et al. [15] analyzed the QT (ventricular depolarization and repolarization interval) and RR intervals (measures the heartbeats rate between two cardiac cycles) of ECG signals and ECG derived respiration combined with a machine learning-based classifier, specifically the SVM, for stress detection in automobile drivers [21]; but, in this study, the authors concluded that the more features are used, the more accurate the methodology. This condition makes the implementation in real time more difficult and it may take more time to process for delivering the stress alert if an episode is detected. Wang and Guo [16] proposed an autoencoder classification model for driving stress recognition using ECG, HRV, foot (FGSR), and hand galvanic skin response (HGSR) signals. The obtained results show that their proposal combines the four signals for determining stressed automobile drivers with an accuracy of 97%. Chui et al. [17] combined the convolution and cross-correlation methods with a multiple-objective genetic algorithm optimized deep multiple kernels learning SVM for recognizing automobile drivers stressed by using ECG signals, achieving an accuracy of 96.9%. Despite the obtained results, the authors mention that their proposal presents a high computational load, which would limit its application in real-time due to their long delays in the diagnosis. They reported that an accuracy of 98% is reached, distinguishing both groups. Recently, Cruz et al. [18] investigated three features of ECG signals (i.e., ECG-derived Respiration, Respiration Rate, and QT interval) fused with the wavelet transform and machine learning based on SVM (used as classifier) for identifying automobile drivers with stress, reaching an accuracy of 96.3%. On the other hand, Wang et al. [19] acquired 10 signal sets which include ECG, EDA, and breathing rate signals, the authors used two wearable devices to obtain these signals. They used four signals: (1) Heart Rate (HR), (2) Breathing Rate (BR), (3) HRV, and (4) Galvanic Skin Response, from which four statistical features were extracted (mean, median, first and third quartile). In this study, a preprocessing step was employed, consisting of applying a low-pass filter and a change point detection algorithm. Once this step was executed, a convolutional neural network was used to detect stress in drivers, obtaining an accuracy of 89% using the leave-one-out validation. Zontone et al. [20] developed a methodology based on EDA and ECG signals to detect stress or no stress in car drivers. The authors obtain the ECG signal and hand EDA signal, eliminating the motion artifacts; next, the data are sent via a wireless connection to a computer. For the ECG signal, eight features were extracted, such as RR intervals, standard deviation of RR intervals, mean value of HR, and HR mean derivative value, among others. From the EDA signal, only five features were extracted, including energy, mean absolute value, mean absolute derivative, and max absolute derivative. Once the features were obtained, they were normalized for developing both SVM classifier and an Artificial Neural Network with a single input layer, two hidden layers, and a single output layer. The authors report 76.57% and 77.59% accuracy for each classifier, respectively. Despite obtaining promising results in the aforementioned works, there are some limitations that may be discussed: (1) a high computational cost, which can restrict the stress detection in real-time, and (2) they require the combination of diverse physiological signals. In addition, it is important to mention that most of these studies have been focused on analyzing ECG and EDA (electrodermal activity) signals, which can present diverse problems. For instance, the ECG signals can be affected by several heart conditions such as arrhythmias [22]; on the other hand, EDA signals are strongly affected by sweat generated, which depends on the area of the body that is being studied [23]. In this regard, EMG signals can offer an alternative to detect automobile drivers with stress since it presents several advantages in comparison with those mentioned above. For example, (1) EMG signals are safe, easy, and noninvasive sources of information, (2) they are capable of detecting changes in a particular muscle because of the correlation between the biochemical and physiological changes during the movements of the muscle, and (3) they are capable of isolating the muscles needed in the study (as related with stress condition) without the interaction of the nearby muscles or other muscles [24,25]. Nevertheless, the identification of relevant features into the EMG signals represent a challenging task in associating them with stress detection [14]. For example, Katsis et al. [26] investigated diverse nonlinear measurements of EMG signals (i.e., root mean square and mean value) as well as ECG, EDA, and respiration signals with a SVM for detecting automobiles drives with stress, reaching 79.3% accuracy. Fu and Wang [27] integrated two nonlinear measurements (peak factor and maximum of cross-relation curve) with an SVM for recognizing automobile drivers with stress by using EMG and ECG signals. The authors report an accuracy of 86.7% for distinguishing automobiles drivers with stress. Recently, Wang and Guo [16] combined a multilayer representation learning module and an ensemble classification module under the AdaBoost framework for distinguishing automobile drivers with stress using EMG signals. They mention that their proposal is capable of recognizing stressed automobile drivers with an accuracy of 58%.

On the other hand, it should be noted that with the development of deep learning frameworks, there is a new and updated version of machine learning [12], different applications for detecting pedestrians [28] or processing medical images for detecting mammographic lesions [29] have been recently proposed. The former uses a body part-detector for a convolutional neural network-based classifier, reporting a reduction of the misclassification error of about 10%, whereas the latter used a modified version of the VGG neural network to perform the lesion detection using contrast, patient information, texture, and geometrical features, obtaining an area-under-the-curve (AUC) of 0.94 (the closer to 1, the better the classifier). Considering these promising results, Rastgoo et al. [30] developed a convolutional neural network combined with a long short-term memory network to fuse an ECG with the brake, steering wheel, and gas pedals signals, as well as environmental data including distance from other vehicles and time of day for detecting stress in automobile drivers. They obtained an accuracy of 92.8%. The authors of these works indicate that their proposals require a significant amount of training data to generate a methodology with a reasonable generalization capability. In this sense, data augmentation, which is the procedure of generating new data training by slightly modifying the original data [31], is used. Yet, to modify physiological signals, it is necessary to have a model, which in most cases is a challenging task due to the highly nonlinear nature of human organs [32]. Considering that the most effective methodologies for detecting stress fuse the information of more than one physiological signal or, if a deep learning framework is employed, data augmentation is used to increase the database size, the necessity of developing methodologies that do not require the fuse of features extracted from physiological signals nor the increase in the database size remains an opportunity area. These methodologies, based on low-complex processing techniques, can detect features using EMG signals for achieving a high accuracy and a low-computational use classifier, allowing drivers take preventive actions in order to reduce their chances of having a car accident.

In recent years, statistical time features (STFs) have demonstrated to be efficient for predicting seizures [33], evaluating the health condition of civil structures [34], induction motors [35], diagnosing neurodegenerative diseases [36], evaluating sleep disorders [37], and detecting alcohol use disorders [38], among other applications. They present great advantages such as low complexity, low computational costs, and are capable of measuring changes in non-stationary time signals [37], such as EMG signals, which can be useful for identifying relevant features into EMG signals in order to associate them to detect automobile drivers with stress.

In this paper, a new methodology based on machine learning, including STFs and an SVM classifier, is presented for detecting automobile drivers who are experiencing stress using EMG signals. The methodology investigates the potential of 17 STFs (i.e., Root Mean Square (RMS), Shape Factor with RMS (SFrms), Square Mean Root (SMR), Shape Factor with SMR (SFsmr), Crest Factor (CF), Impulse Factor (IF), Latitude Factor (LF), Range (R), Mean (M), variance (Var), Standard Deviation (STD), Skewness (Sk), Kurtosis (K), 5th Moment, (5Mo), 6th Moment (6Mo), Median (Me), and Mode (Md)) for identifying relevant features or patterns in the EMG signals. Then, the calculated STFs are evaluated through the Kruskal–Wallis statistical analysis method (KWM) for determining the most discriminant STFs values. Once these values are selected, an SVM classifier is employed for determining stressed drivers automatically. In order to perform a comparative analysis between different machine learning techniques, an SVM classifier is tested with diverse kernels for getting the highest accuracy possible, and a multilayer perceptron (MLP) is also investigated, where SVM demonstrated to be the most efficient. It should be pointed out that this classifier is selected because SVM can be trained using few samples [39]. The proposal effectiveness is validated using EMG signals acquired experimentally from 10 participants with diverse levels of stress [10].

## 2. Materials and Methods

### 2.1. Employed Dataset

For evaluating the capability of the proposed method for detecting stress in automobile drivers, the EMG signals from an open access database called *Stress Recognition in Automobile Drivers*, available at Physionet (https://physionet.org/content/drivedb/1.0.0/, accessed on 28 April 2021) provided by Healey and Picard [10], are used. The database was originally composed of 24 EMG signals (participants), but the number of participants in the database has suffered a reduction over the years (no reason is offered by database administrators about the reduction of EMG signals). Nowadays, the database contains the EMG signals of 17 participants; however, only 10 participants present full information of EMG signals [40], which are employed in this work. Table 1 presents the duration of EMG signals for each participant, which vary from 65 to 93 min. 

**Figure 2:**
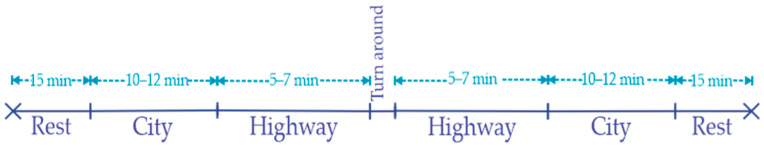
Description of the driving path.

In order to ensure the drivers were exposed to different levels of stress, they followed a driving protocol or route that imitates typical driving scenarios. In addition, the participants were asked to respect the speed limits and to turn off the radio [10,15,37]. The driving route includes diverse scenarios: rest, city driving, and highway driving periods (see Figure 2), which were intended to produce in the drivers low, medium, and high levels of stress, respectively. Furthermore, the driving route also included a scenario called “turn around”, which indicates that the drivers had to drive back following the same route in the opposite direction; the only difference was in the highway driving because drivers were asked to stay in the second to right lane until they saw the exit sign since the right lane leads to an exit [36]. In the rest scenario, the participants were relaxed with closed eyes and the car was in an idle state for about 15 min. In the city scenario, the drivers were on a main street with traffic lights and faced unexpected risks generated by cyclists or pedestrians for 12–15 min. In the highway scenario, the drivers drove between 7–10 min in straight lanes with light traffic in the midmorning or midafternoon traffic; the times varied depending on traffic and the driver [10,18,39]. In order to corroborate the stress level experienced by each participant, two questionnaires were applied immediately after the subjects completed the route, which are described by [10] in detail. 

### 2.2. Preparation of the EMG Signals

**Figure 3:**
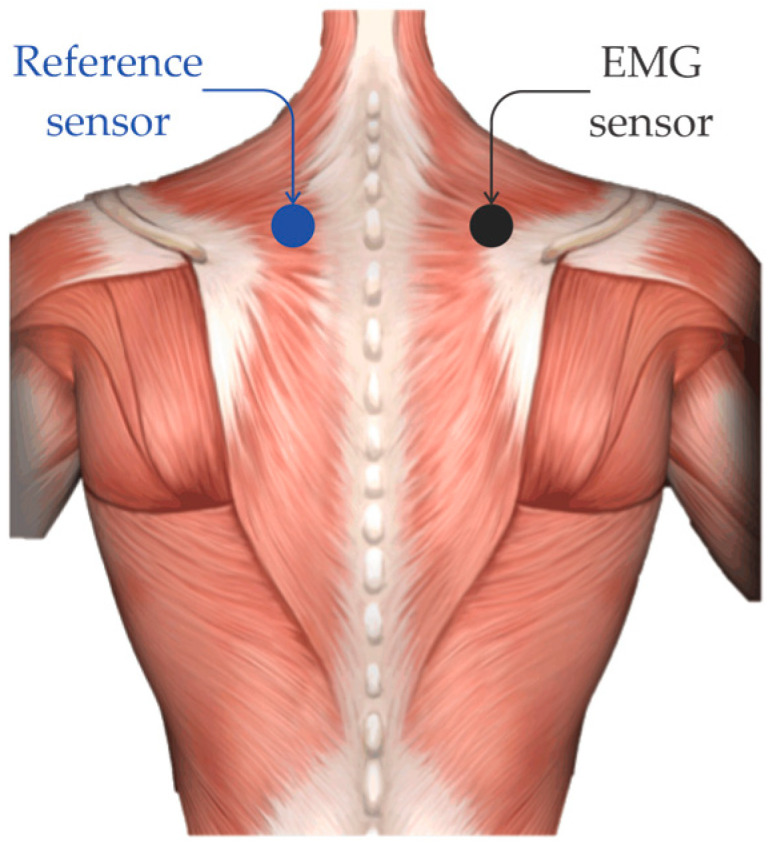
Localization of the EMG sensor in subjects.

**Figure 4:**
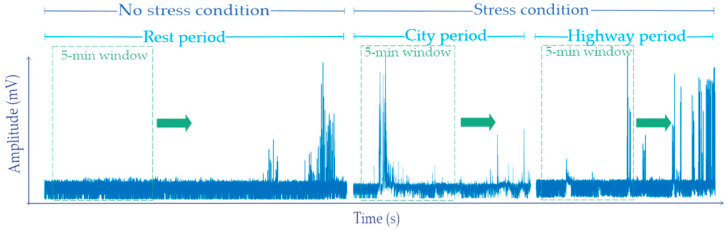
Extracted EMG signal segments for each scenario.

For measuring the EMG signals of participants, two sensors (or electrodes) were employed. One was placed on the trapezius muscle (shoulder) in the right side and the other one was used as a reference sensor (see Figure 3). The sensors were connected to a FlexComp analog-to-digital converter placed at the trunk of the car, so the drivers had full visibility and were not distracted. The data were transmitted by a fiber optic cable and the subjects were isolated from the electrical system of the car in order to prevent any electric shock. The FlexComp unit was connected to the modified car’s computer used for this experiment [10]. For acquiring the EMG signals, a sampling frequency of 15.5 Hz was employed because the converter saves samples rates in multiples of 31 samples per second [10]. In this study, five segments were extracted from the EMG signals. One segment corresponds to the first rest period, which was used as a baseline since the driver was relaxed and had not been exposed to any driving stress [10], and the other four segments correspond to the two city and highway periods, respectively. A total of 50 segments of information were extracted (5 segments for each EMG signal) for validating the proposed methodology. It is important to mention that each extracted segment or time window contains 5 min of information because this time window has shown to be adequate for capturing relevant information in physiological signals such as EMG signals [10,41,42,43]. On the other hand, in this work, the obtained segments of the rest period are considered as a no stress condition (NSC), while the segments of city and highway are employed as the stress conditions (SC) because they can compromise a normal conduction [18]. Figure 4 shows the EMG signal behavior for the three scenarios; it is possible observe that significant differences among the three scenarios cannot be distinguished. Hence, a method with the capability of identifying reliable features in these signals for recognizing if an automobile driver has stress or not is of imperative importance.

## 3. Methodology

**Figure 5:**
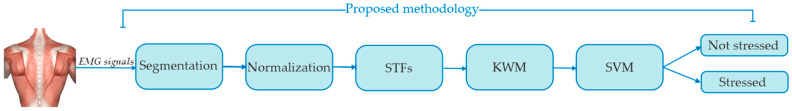
Schematic diagram of the proposed methodology.

Figure 5 graphically presents the proposed methodology based on machine learning, including STFs and an SVM classifier, for stress detection in automobile drivers. Firstly, the EMG signal of each participant was segmented or divided into 5-time windows, each one 5 min long: 1 segment of rest period, 2 segments of city period, and 2 segment of highway period, resulting in 50-time windows or segments considering the 10 participants. Then, each time window was normalized in order to obtain the same magnitude for all the analyzed segments. Once the segments or time windows are normalized, they were processed by means of 17 STFs in order to find the features with the capability of recognizing automobile drivers with stress. Then, the STFs values, obtained in the previous step, were evaluated by KWM for determining the most useful feature(s) for driving stress recognition. Finally, the most discriminative features, selected according to the KWM, are employed for the training machine learning-based classifiers, SVM and MLP, for automatically determining the drivers’ condition (stressed or not stressed), as seen in Figure 5. It should be pointed out that the explainability of the machine learning algorithm allows for generating a deeper comprehension of the model’s efficacy and what model should be selected depending on the features used in its development [13,40]. In this sense, since SVM has different kernels that can be trained, they are employed to find out which one has the best efficacy using the STF as feature inputs and a training strategy; thus, information about model explainability can be determined.

### 3.1. Normalization

Generally, the measured EMG signals for each participant can present different scales or magnitudes because they can be affected by electrode configuration and placement, skin preparation and impedance, perspiration and temperature, and the number of fibers or blood flow in the muscle, among other factors [44]. Hence, it is necessary to normalize the EMG signal for each participant in order to obtain the same scale or magnitude for each test. In this regard, the EMG signals and segments are normalized by subtracting the mean µ from the raw signal and dividing it by the deviation standard σ, as follows [18]:

(1) $$\tilde{x}=\frac{x-\mu }{\sigma }$$

### 3.2. Statistical Time Features

STFs have demonstrated to be reliable tools for recognizing patterns or features in time signals with non-stationary properties, which have allowed for evaluating the health condition of structures [34] and induction motors [35], as well as predicting [33] and detecting diseases [36,37], among other applications. In general, STFs allow for measuring properties such as the range, asymmetry, convergence, and dispersion, among others, of time signals without needing to transform them into another domain [40,41]. Hence, they present a low computational load and complexity, which can be suitable advantages for recognizing stress in automobile drivers in a timely manner [34]. The 17 STFs investigated in this paper for discovering reliable features into the segmented EMG signals are described as follows:

(2) $$\mathrm{Mode}\;=\;\mathrm{MO}\;=\;L_{i}\;+\;c\frac{d^{-}}{d^{-}+d^{+}}$$

(3) $$\mathrm{Mean}\;=\;M\;=\;\frac{1}{N}\sum_{i=1}^{N}x_{i}$$

(4) $$\mathrm{Range}\;=\;R\;=\;x_{imin}-x_{imax}$$

(5) $$\mathrm{Variance}\;=\;\mathrm{Var}\;=\;\frac{1}{N}\sum_{i=1}^{N}{\left(x_{i}-M\right)}^{2}$$

(6) $$\mathrm{Standard}\;\mathrm{Deviation}\;=\;\mathrm{STD}\;=\;{\left(\mathrm{Var}\right)}^{\frac{1}{2}}$$

(7) $$\mathrm{Impulse}\;\mathrm{Factor}\;=\;\mathrm{IF}\;=\;\frac{x_{imax}}{\frac{1}{N}\sum_{i=1}^{N}\left|x_{i}\right|}$$

(8) $$\mathrm{Square}\;\mathrm{Mean}\;\mathrm{Root}\;=\;\mathrm{SMR}\;=\;{\left(\frac{1}{N}\sum_{i=1}^{N}x_{i}{}^{\frac{1}{2}}\right)}^{2}$$

(9) $$\mathrm{Shape}\;\mathrm{Factor}\;\mathrm{Square}\;\mathrm{Mean}\;\mathrm{Root}\;=\;\mathrm{SFsrm}\;=\;\frac{\mathrm{SMR}}{\frac{1}{N}\sum_{i=1}^{N}\left|x_{i}\right|}$$

(10) $$\mathrm{Root}\;\mathrm{Mean}\;\mathrm{Square}\;=\;\mathrm{RMS}\;=\;{\left(\frac{1}{N}\sum_{i=1}^{N}x_{i}{}^{2}\right)}^{\frac{1}{2}}$$

(11) $$\mathrm{Shape}\;\mathrm{Factor}\;\mathrm{Root}\;\mathrm{Mean}\;\mathrm{Square}\;=\;\mathrm{SFrms}\;=\;\frac{\mathrm{RMS}}{\frac{1}{N}\sum_{i=1}^{N}\left|x_{i}\right|}$$

(12) $$\mathrm{Crest}\;\mathrm{Factor}\;=\;\mathrm{CF}\;=\;\left|\frac{x_{imax}}{\mathrm{RMS}}\right|$$

(13) $$\mathrm{Latitude}\;\mathrm{Factor}\;=\;\mathrm{LF}\;=\;\left|\frac{x_{imax}}{\mathrm{SMR}}\right|$$

(14) $$\mathrm{Skewness}\;=\;\mathrm{Sk}\;=\;\frac{1}{N{\left(\mathrm{RMS}\right)}^{3}}\sum_{i=1}^{N}{\left(x_{i}-M\right)}^{3}$$

(15) $$\mathrm{Kurtosis}\;=\;k\;=\;\frac{1}{N{\left(\mathrm{RMS}\right)}^{4}}\sum_{i=1}^{N}{\left(x_{i}-M\right)}^{4}$$

(16) $$5\mathrm{th}\;\mathrm{Moment}\;=\;5\mathrm{Mo}\;=\;\frac{1}{N{\left(\mathrm{RMS}\right)}^{5}}\sum_{i=1}^{N}{\left(x_{i}-M\right)}^{5}$$

(17) $$6\mathrm{th}\;\mathrm{Moment}\;=\;6\mathrm{Mo}\;=\;\frac{1}{N{\left(\mathrm{RMS}\right)}^{6}}\sum_{i=1}^{N}{\left(x_{i}-M\right)}^{6}$$

(18) $$\mathrm{Median}\;=\;\mathrm{Me}\;=\;x\left(\frac{N+1}{2}\right),if\;N\;is\;odd\;number\;\mathrm{or}\;\frac{1}{2}\left(\left(x\frac{N}{2}\right)-\left(x\frac{N}{2}+1\right)\right),\;if\;N\;is\;even\;number$$

where *x_i_* represents the time signals from *i = 1,2,3..., N*, *N* is the number of data points, *L_i_* corresponds to the lower limit of modal class, *c* is the width of the modal class, and *d^−^* and *d^+^* are the absolute differences of the modal interval and the classes of the neighbor intervals, respectively. It is important to mention that the investigated STFs measure different characteristics in the time signals can or cannot provide suitable information to perform a correct diagnosis. For this reason, it is necessary to employ a statistical method for measuring the relevance of each STF in order to identify the most useful features for recognizing automobile drivers with stress in an accurate manner. In this regard, the Kruskal–Wallis method (KWM) was used for this step. This method can be used regardless of the probability distribution the database has [33,34], unlike the analysis of variance (ANOVA) method, which should be used with data that have a Gaussian (normal) distribution. Physiological data are known for having a non-Gaussian distribution [33]; hence, methods such as KWM are adequate to be used for this type of data.

### 3.3. Kruskal-Wallis Method

KWM is a non-parametric method employed to estimate the statistical independence of datasets with non-normal distributions [45,46]. It has been employed for determining the most suitable features for detecting or estimating seizures [33], drowsiness levels [47], and respiration [48], among other applications. KWM evaluates the variation between datasets and the variations within each dataset [49] by calculating a probability value (*p*-value). In addition, a null hypothesis value is established (generally 0.01), which indicates that the median of both groups is equal [45,50]. In this sense, if the *p*-value is smaller than the null hypothesis value, the null hypothesis is rejected, indicating that the selected datasets and the remaining datasets can be differentiated. On the other hand, if the *p*-value is higher than the aforementioned range [51], the null hypothesis is accepted, indicating that the datasets have similar information and, therefore, they cannot be used for differentiating between groups (i.e., stress and no stress). Hence, the *p*-value is used in this work to determine which features are the most discriminant for recognizing automobile drivers with stress.

### 3.4. Support Vector Machine

SVM, a classification method, is a supervised learning algorithm that assigns labels to the objects of each class [52]. SVM maximizes a mathematical function based on the data through the use of a hyperplane and maximum margins defined by the support vectors (the closest points of data to the hyperplane), which are lines that separate the data in two sets: “positive” or “negative” [39,53]. To perform this task, SVM uses a kernel to project the data in a higher dimension space [49], thus obtaining an efficient classifier. The most common kernels are linear, Gaussian, and radial basis function (RBF) [54]. Depending on the employed kernel, SVM can be classified into linear and non-linear SVM. The former is when the data only have two classes and can be divided linearly [55,56]. On the other hand, if the data cannot be separated linearly, the employed data are transformed into a feature space where they can be linearly separable [57]. In this regard, SVM can be classified as shown in Table 2, where *P* is the number of predictors [54].

SVM has been widely used for classification due to their high accuracy, robustness, parametrization by means of kernel functions, and for being capable of analyzing large datasets [51,56,57]. Therefore, in past decades, this algorithm has been applied in biomedical applications such as stress recognition [26], breast cancer image classification [52], classification of elbow EMG signal [58], and classification of hand arthritis stages [59], among others. Considering all the aforementioned benefits, its use is investigated, including all mentioned kernels, in this work for differentiating between automobile drivers with stress and without stress automatically.

## 4. Results

**Figure 6:**
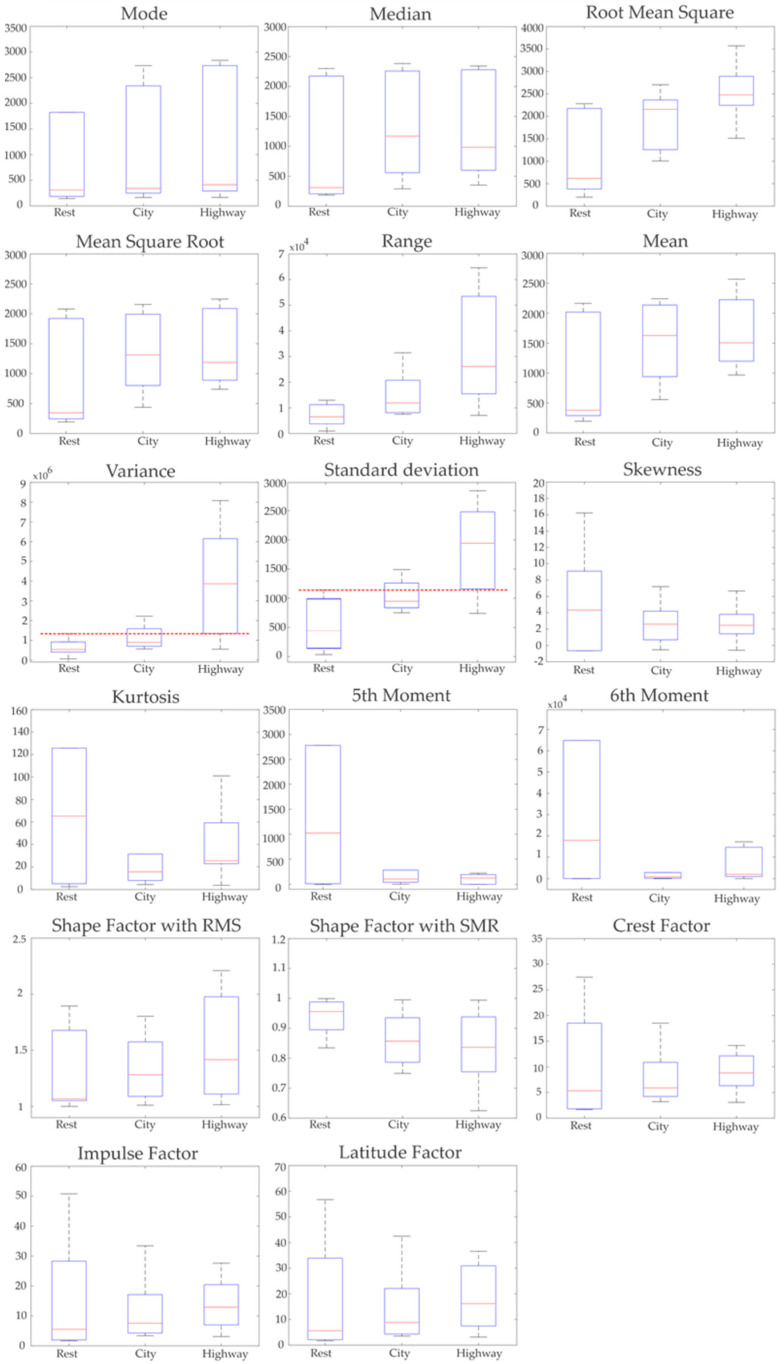
Box diagrams of the 17 STFs of the three periods without normalization.

Using the steps of the proposed methodology, five segments of the EMG signals were extracted, each of them lasting 5 min. In total, 50 segments were extracted from the 10 drivers’ signals. Once the signals were segmented, they were analyzed using the 17 STFs to obtain the features that allow the detection of a stress event in drivers. Considering the 50 segments, 850 features were calculated, making it necessary to perform the statistical test to find out which ones can contribute to developing an efficient classifier for efficiently recognizing automobile drivers with stress. In order to perform this task, the KWM was employed for determining the most reliable features with the capability of distinguishing between an automobile driver with and without stress. It is worth noting that MATLAB was employed to implement the proposed methodology. Figure 6 shows the box plots for all the STFs without the normalization procedure. It can be seen that most of the features are completely overlapped among all the scenarios, making the development of an effective classifier a particularly challenging task. However, the variance and standard deviation present a lesser overlap than the other 15 STFs, the obtained values for both STFs are still overlapped more than 50% between the rest and city scenarios (denoted by dotted red lines in Figure 6), limiting their usefulness for discriminating between a person with and without stress. Hence, these results are expected since the magnitudes of all the STFs have different values, leading to extreme values that make difficult the direct comparison between them [60]. One possible solution is the normalization of the segments, as is proposed in this article, in order to obtain more regular and uniform data without considering the magnitude each variable has [60].

**Figure 7:**
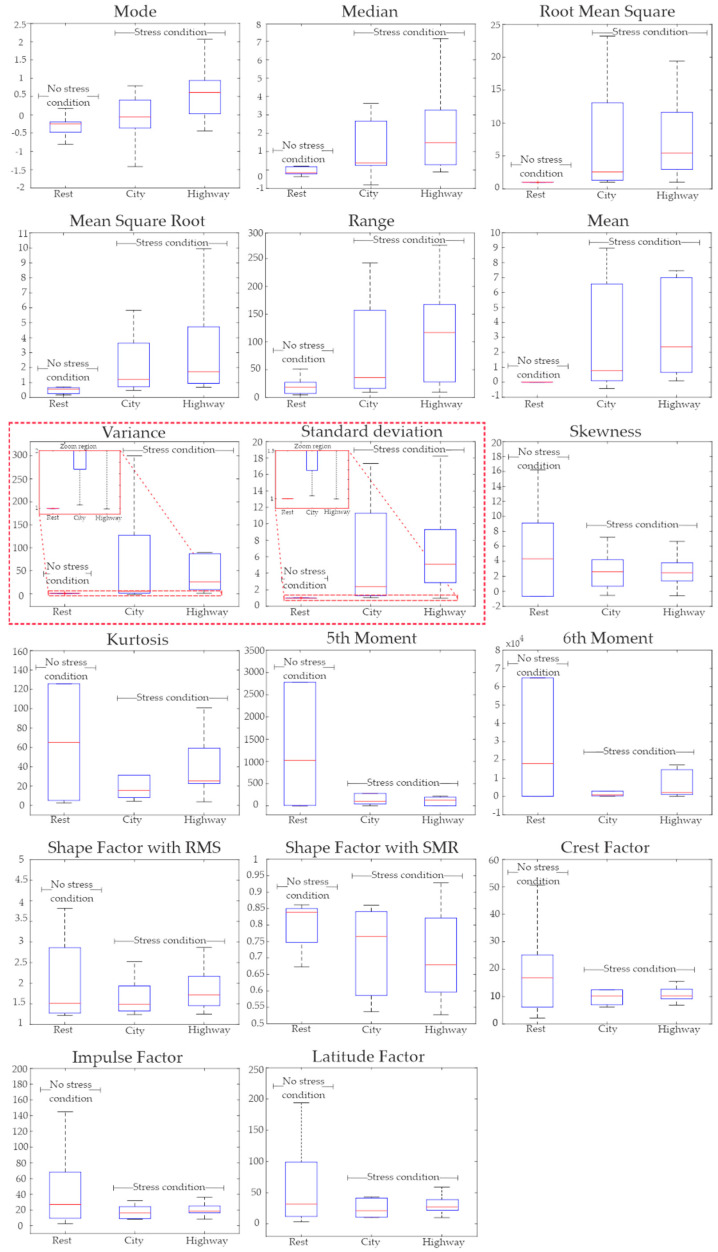
Box diagrams of the 17 STFs of the three periods with the normalized segments.

Considering the abovementioned concepts, the normalization procedure was applied in order to have segments with magnitudes that can be directly compared regardless of their original scale. For this purpose, each segment was normalized by using their rest period, as is described in Equation (1). Figure 7 illustrates the data distribution estimated by all the STFs for the three periods (rest, city, and highway) using the normalization procedure. From this figure, it can be seen that most of the STFs have significant overlapping zones between rest, city, and highway scenarios, limiting its utilization as inputs to the classifier. However, it is worth noting that the variance and standard deviation box plots (denoted by the red trace) have the lowest overlapping zones among all the STFs. It is also worth noting that that these zones are smaller than those obtained without the normalization procedure (see Figure 6), as demonstrated in the zoom region (see Figure 7). In consequence, the normalization procedure allows the enhancing of the subtle differences that the EMG signals have due to the presence of a stress episode, which is masked by the extreme amplitude values that each segment has [60].

**Figure 8:**
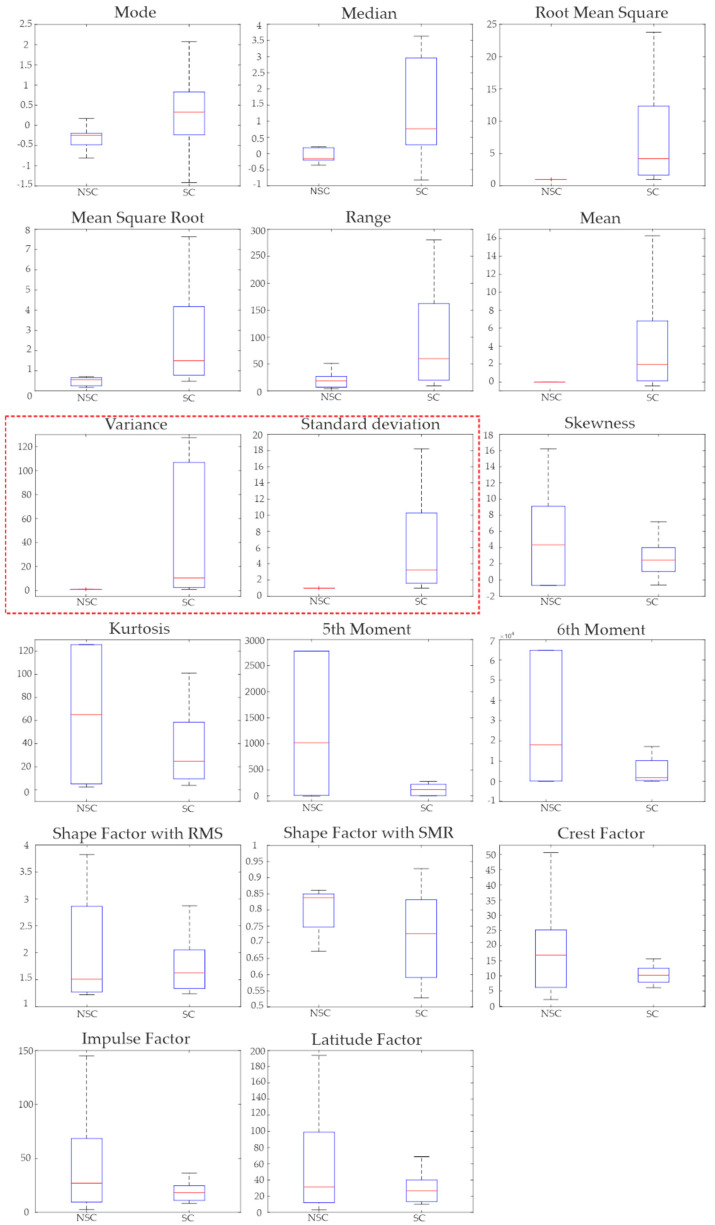
Box diagrams of the 17 STFs of no stress condition (NSC) and stress condition (SC).

On the other hand, a closer inspection of the results of both the variance and standard deviation box plots indicates that both features can potentially be used to detect stress events regardless of severity, which is a highly desirable feature. To confirm this fact, the STF values are joined to generate two categories: no stress (rest) and stress (city and highway). Then, KWM is reapplied to graphically depict the distribution of the values in the box plots shown in Figure 8. It should be pointed out that this procedure is employed for all the STF in order to confirm the results presented in Figure 7. From Figure 8, it is observed that, except for the variance and standard deviation, all the STF have significant overlap zones, confirming the results presented in Figure 7. On the other hand, the box plots for variance and standard deviation STF (highlighted by the red trace) do not present a significant overlap between them. This result allows for reaffirming the potential they have as inputs to the SVM classifier.

It should be noted that both variance and standard deviation measure data spread, either by each sample with its corresponding mean (variance) or the variability of the samples within the same category (standard deviation). In other words, the variance measures the amount of amplitude variability that the samples can reach, and the standard deviation indicates the degree of dispersion that each value can have within all the measured samples [61]; hence, both STFs complement the measure of the data spread [61]. For these reasons, in this paper, the variance and the standard deviation are determined as the most significant features to detect a stress event in drivers, and they can be used as inputs to the classifier. 

The abovementioned results are confirmed by the *p*-values depicted in Table 3, where it is seen that the variance and standard deviation have the lowest *p*-values of all the STF. This confirms the hypothesis described above and further confirms the results depicted in Figure 7 and Figure 8. Although the median and SMR have a low *p*-value, they have a slight overlap between the rest and city scenarios, as seen in Figure 7, which is more evident when the stress and no stress scenarios are considered (Figure 8). This overlap generates a decrement in the classification performance, which is an undesirable scenario. In consequence, median and SMR are discarded as potential inputs. 

Once the most discriminative STFs, i.e., variance and standard deviation, have been selected according to the results estimated by KMW and Figure 7 and Figure 8, they are employed for training the machine learning algorithm (SVM classifier) in order to automatically identify the stress condition in drivers. It should be pointed out that SVM offers a reasonable performance when dealing with low-size databases [39], which is the case in this study. For this purpose, the Iterative Single Data Algorithm [61] is used as the training strategy to obtain the classifier parameters. Broadly speaking, this training algorithm formulates the parameters’ estimation as an optimization problem, where a Lagrange dual function is introduced to determine both the upper and lower boundaries (whose geometrical shape are determined by the employed kernel). 

In consequence, the Iterative Single Data Algorithm modifies, at each iteration, one of the Lagrange multipliers of the dual function, allowing the removal of the unchanged values of the other multiplier [61]. The training scheme uses the 10-k-fold-cross. This scheme randomly divides the training data into *k* subgroups with the same number of samples. Next, from the *k* subgroups, one of them is used as the validation data for testing the model, and the remaining *k-1* subsamples are employed as training data (to estimate the parameters the Iterative Single Data Algorithm requires). This procedure is repeated *k* times, where the partial accuracy results are then averaged to generate the overall classifier accuracy. The advantage of this method is that all samples are equally used for both training and validation, but at the same time, each sample is used only one time for validating the model [61]. This paper uses a *k* value of 10 since it provides the best compromise for generating the most possible subgroups without compromising the diversity in the subgroups [62], as this will ensure that all the samples are used both for training and validation. 

**Figure 9:**
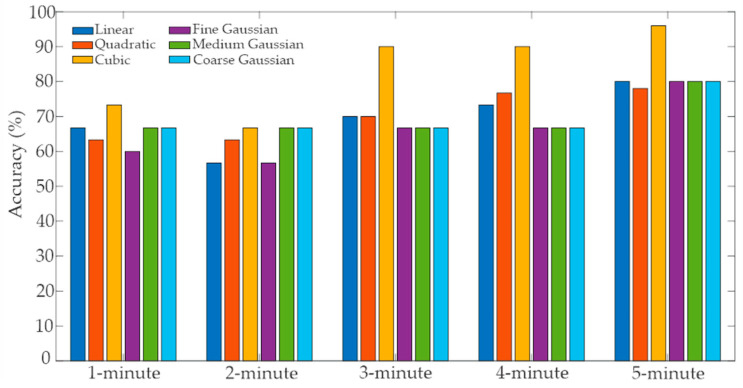
Accuracy for each window length for the SVM classifier.

In this study, all the kernels shown in Table 2 were analyzed to achieve the best possible accuracy. Table 4 and Figure 9 present the accuracy obtained from the abovementioned kernels, studying the size of each segment. It can be seen that as the segment size increases its length, more accurate classification rates are achieved, as the accuracy value reaches above 90% when the segment length is greater than 3 min using the cubic kernel. Interestingly, windows greater than 5 min do not increase the classifier accuracy. Even when one might think that the 3-min window length is sufficient to detect with a reasonable accuracy a stress episode, using only a few more samples generate an increase in accuracy, which is the ultimate goal when designing biomedical signal classifiers. Moreover, it is known that a lower accuracy value also decreases the ability to correctly detect stressed driver (sensitivity) and the non-stressed driver (specificity) [33]. In consequence, the 5-min window length offers the best compromise between an accurate classifier and the always-desirable opportune detection. This length also confirms the results presented by similar works [10,42]. 

On the other hand, it can also be mentioned that the cubic kernel in the SVM offers the highest accuracy since this kernel better captures the geometrical shape of the separation between the stress and no stress scenarios, generating the highest accuracy possible, 96%. Moreover, the obtained values for the sensitivity and specificity are 100% and 95%, respectively. These features are important metrics when dealing with physiological signals-based classifiers, as they indicate the performance to detect a stressed driver (sensitivity) and non-stressed driver (specificity) accurately [33]. The obtained results affirm that the selected STF can deliver a reasonable classifier. In this sense, the model explainability can be resumed as follows: when the features have a low separation between them, non-linear kernels should be selected to find the one that can capture the shape of the boundary that divides the different classes.

In order to make a comparative analysis of different machine learning techniques, the SVM algorithm and a neural network classifier is designed. In this case, a multilayer perceptron (MLP) is chosen with two neurons in the input layer (the variance and standard deviation), 10 neurons in the hidden layer, where the activation function is the bipolar sigmoid, and 2 neurons in the output layer (stressed or not stressed). The training algorithm used is the Levenberg–Marquardt scheme as it provides the best compromise between the training speed and the classifier accuracy [63]. It should be noted that the same validation procedure, the 10-k-fold-cross validation, is used. The area under the curve (AUC) value is also estimated for both classifiers in order to determine the classifier capability to distinguish between the two scenarios (stressed and no stressed) [64]. The closer the AUC value is to 1, the better the classifier capability to detect, in this work, the stress episodes. Table 5 shows the obtained results

From this table, it is seen SVM has a better accuracy compared with the MLP, as SVM accuracy is higher by a 13% margin; moreover, the SVM has a higher AUC value (0.97) than the MLP classifier (0.84), indicating that the SVM classifier can detect stress episodes more accurately than the MLP. In this sense, it should be noted that MLP decreases its performance when processing low-separated data [65]; moreover, this issue is exacerbated since the database size is small [66]. Other classification algorithms, such as random forest and decision trees, are better suited to deal with multiclass classification [40]; evidently, this work is only a binary classifier (stressed or not stressed). In consequence, the obtained result when employing these classifiers to the presented problem might be an unsuitable option. On the other hand, SVM can better handle the samples whose separation is small, as seen in Figure 7 (dashed red lines), since the boundaries that the training algorithm determines are fine-tunned [65].

## 5. Discussion

Table 6 presents the results obtained by the proposal and other methodologies that employ the same dataset, where a brief description of the methods used, the number and type of physiological signals, as well as the accuracy reported by the authors is presented. From this table, it can be noted that the proposal obtains a 96% accuracy using only EMG signals coupled with the STFs, unlike previous works [25,26] that use different types of signals in their methodologies. The obtained results affirm that the proposal can effectively detect stress events in drivers regardless of the scenario in which they are driving.

Detecting stress in drivers has proved to be a difficult task since physiological signals require robust methodologies that can detect and associate the changes in stress conditions generated in drivers. For these reasons, most authors have proposed methodologies that use more than one physiological signal to determine if a driver suffers a stress episode or not [25,26,40], achieving reasonable accuracies of 79%, 86.7%, and 81%, respectively. It should be pointed out that these methodologies use algorithms that require a significant amount of time to deliver a result, impeding the continual evaluation of the windows, limiting the real-time operation, which is a desirable feature because the earliest a stress event is detected, the driver can be alerted and take actions to prevent traffic accidents. These results might lead to consider using further information—for instance, information about how the car has been driving [67]—in order to increase the accuracy of the developed classifier, as shown in [30]. Still, it is seen that complex and high-computational algorithms are required to fuse the information and perform the stress detection, which results again in the impossibility to operate the proposal in real-time.

One interesting fact is that the sensors that measure the car information do not require physical connection with the driver [68]. In this sense, its utilization shows that the development of novel sensors that can measure contactless physiological signals has become an attractive research area, as its utilization can allow the miniaturization of signal acquisition systems, resulting in the massification of these systems with the benefits of detecting dangerous stress levels that drivers must avoid.

On the other hand, when only the EMG signal is processed, methods that are complex and have high-load computational burden in order to filter and classify the detected changes are employed [18], obtaining efficiency lower than 60%. Evidently, these types of methodologies cannot be implemented for real-time detection strategies nor can they detect the changes produced in the EMG signal. On the contrary, the proposed method obtains 96% accuracy using a simple-yet-effective methodology that can detect the changes produced in the EMG signal regardless of the stress severity based on the estimation of STF. It is important to mention that the performance of this methodology is higher compared with other works since the accuracy is almost 10% higher. This parameter was used in generalization performances in [69,70], and the obtained classification error is only 2% of the data used for training and validate the methodology, which is highly desirable for a future implementation. SVM has proven to be an effective classifier with a stronger generalization performance than traditional neural networks [71].

The proposal has some advantages: (1) only one physiological signal is employed; (2) no preprocessing step is used, which is very convenient feature since the computational burden is reduced; and (3) the implementation of the SVM classifier can be done in an efficient way as it requires only a third-degree polynomial equation, whose implementation only requires sums and multiplications, which are mathematical operations that are computer-time optimized in a digital signal processor. In consequence, a real-time operation can be achieved, making stress detection fast and allowing drivers to take precautions to avoid car accidents, as well as reducing stress-related health problems in the population. However, it is important to (1) create and evaluate a new EMG signal database with a larger signal to corroborate this methodology; (2) develop new methodologies capable of detecting between the three stress levels mentioned by the authors in [5] in order to have different alerts depending on the stress level; (3) develop deep learning algorithms that require less computational burden in order to generate methodologies that can benefit from the advantages that this type of strategy has, including enhanced generalization capabilities even when dealing with low-size databases; and (4) explore novel algorithms for data augmentation for creating different signals that can represent information of more drivers, as this will allow to create a greater database and, if necessary, to perform model patching procedures in order to incorporate missing information that the original database does not have.

## 6. Conclusions

When any person is subjected to any type of threat, the sympathetic nervous system reacts and generates physiological and chemical changes to the body, inducing a sudden release of hormones, which generates a stress episode [1]. Since it is well known that stress in drivers is one of the main reasons behind car accidents, this paper investigates the capabilities that STF can have in detecting subtle differences in EMG signals that different stress scenarios produce in car drivers. KWM is used as the method that detects the statistical independence of each set of STF values to determine the ones that have the most discriminant condition to be used as the inputs to a classifier that can determine if a driver is stressed or not; in this study, different machine learning algorithms such as SVM and MLP were compared.

The presented results show that normalization is required to ensure the best possible results when dealing with features that have a different variation range. Moreover, variance and standard deviation are the most discriminant STFs as they do not have significant overlapped zones between the stressed and not stressed conditions. This indicates that they are effective in detecting and quantifying the subtle changes the stress condition generates in EMG signals, allowing the use of an SVM classifier with 96% accuracy. Compared with methodologies that use either one or more physiological signals, the proposal significantly improves the presented results with a simple yet effective alternative since it does not require preprocessing algorithms nor complex classifiers. These features make the proposal an attractive option for systems that can operate in real-time environments since continuous alerts can be generated, which can reduce all the associated consequences that a car accident may have. To confirm these results, the proposal should be explored with a bigger database either by testing more drivers or using data augmentation algorithms, such as the autoencoder framework. Also, improvements based on explainable machine learning techniques should be done in order to assess the stress level, since this can personalize the alert severity that a re-al-time system can deliver, which might reduce even more the personal and economic consequences that a car accident can have [72,73]; Furthermore, the development of an updated database that can consider all the technological developments that are now present in the car and the actual driving scenarios is necessary, as the driver workload could be unnoticeably increased, hence generating additional stress initiators such as fatigue [68] or cognitive overload [74] that may not be related to the activity of driving but can increase the stress the driver feels. In this sense, the proposal’s effectiveness needs to be tested, as a recalibration procedure may be required in order to deliver the best possible results. Moreover, the development of portable physiological monitoring systems that do not introduce a sense of discomfort are required, i.e., wearable devices in some parts of car such as the steering wheel, seat belts, and seats, as they might further enhance the stress the driver can develop. The development of these research areas can conduce to the massification of stress detection systems with the benefits that this type of systems has. 

## Figures and Tables

**Table 1:** EMG signals duration for each participant.

Participant	Duration (Hours: Minutes: Seconds)
1	1:24:15.9
2	1:20:46.6
3	1:28:38.8
4	1:21:11.5
5	1:10:52.3
6	1:21:16.9
7	1:21:13.1
8	1:23:04.3
9	1:20:28.1
10	1:04:57.9

**Table 2:** Types of SVM and their kernels [51].

Type	Kernel Used
Linear	-
Quadratic	Quadratic
Cubic	Cubic
Fine Gaussian	RBF scale set to P4
Medium Gaussian	RBF scale set to $\;\sqrt{P}$
Coarse Gaussian	RBF scale set to $\;4\sqrt{P}$

**Table 3:** *p*-values obtained using KWM.

Statistical Feature	*p*-Value
Mode	0.0013
Median	7.04 × 10^−6^
RMS	6.21 × 10^−4^
SMR	6.37 × 10^−7^
Range	0.0017
Mean	2.61 × 10^−6^
Variance	1.11 × 10^−6^
Standard deviation	1.11 × 10^−6^
Skewness	0.42
Kurtosis	0.55
5th Moment	0.16
6th Moment	0.42
Shape factor w/RMS	0.91
Shape factor w/SMR	0.02
Crest factor	0.23
Impulse factor	0.39
Latitude factor	0.55

**Table 4:** Obtained accuracy for each kernel.

SVM Kernel	Accuracy (%)				
	1 Min	2 Min	3 Min	4 Min	5 Min
Linear	66.7	56.7	70	73.3	80
Quadratic	63.3	63.3	70	76.7	78
Cubic	73.3	66.7	90	90	96
Fine Gaussian	60	56.7	66.7	66.7	80
Medium Gaussian	66.7	66.7	66.7	66.7	80
Coarse Gaussian	66.7	66.7	66.7	66.7	80

**Table 5:** Comparison between classifiers.

Classifier	Accuracy	AUC
SVM	96%	0.97
MLP	83.3%	0.84

**Table 6:** Comparison with similar works.

Author	Signals	Methodology	Accuracy
Katsis et al. (2008) [26]	EMG, ECG, EDA and Respiration	Two statistical features were extracted as mean value and root mean square. 10-fold-cross validation and SVM classifier was used.	79.3%
Fu and Wang (2014) [27]	EMG and ECG	A preprocessing step was headed with the Fast Independent Component Analysis from both signals. Two nonlinear measurements were obtained from the windows (peak factor and maximum of cross-relation curve). 10-fold-cross validation and Mahalanobis distance used as classifier.	86.7%
Wang and Guo (2020) [16]	EMG	Pseudoinverse Learning Algorithm based Autoencoder (PILAE) was used for the representation learning of signals and AdaBoost classifier was used as the final step. Leave-One-Out-cross validation was employed.	58%
Rastgoo et al. [30]	ECG, vehicle and environmental data	Fusion of the CNN and LSTM models to develop the classifier. CNN is used to fuse the information obtained from ECG, vehicle, and environmental data. LSTM is used as classifier.	92.8%
El Haouij et al. [40]	EDA	4-level Discrete Wavelet Decomposition is performed to the right-hand EDA signal. Haar Wavelet is used as mother wavelet. Random Forest classifier is used.	81%
Proposal	EMG	Statistical Properties are used as features. Support Vector Machine is used as the classifier. 10-fold-cross validation is employed.	96%

## Data Availability

Authors did not collect data from humans or animals. Data used in this research are from publicly available sources (Physionet databank) (https://physionet.org/content/drivedb/1.0.0/, accessed on 28 April 2021), supported by the National Institute of Biomedical Imaging and Bioengineering under grant number R01EB030362.
